# Evaluating the impact of contrast agents on micro and nano mechanics of soft-to-hard tissue interface

**DOI:** 10.1038/s41598-025-12729-6

**Published:** 2025-08-02

**Authors:** Atousa Moayedi, Katerina Karali, Markus Boese, Jurgita Zekonyte, Jovana Radulovic, Gordon Blunn

**Affiliations:** 1 School of Electrical and Mechanical Engineering, University of Portsmouth, Portsmouth, UK; 2 School of Medicine, Pharmacy and Biomedical Sciences, University of Portsmouth, Portsmouth, UK; 3 Carl Zeiss Microscopy GmbH, Oberkochen, Germany

**Keywords:** Mechanical engineering, Tissues

## Abstract

Transmission of strain across the tendon-bone interface otherwise known as the enthesis, is crucial to the movement of the skeleton. Imaging the inner structure and understanding the way that strain is transmitted across this interface is crucial to understanding the way it responds to load, how it becomes injured through trauma and how intervention and materials can be used to repair the enthesis after injury. Micro-CT imaging and digital volume correlation (DVC) have been widely used for musculoskeletal biomechanics analysis. However, there are limitations for soft tissue visualization. Contrast agents (CA) are used to address this, but understanding their potential effects is essential to ensure accurate and reliable characterization of musculoskeletal tissues biomechanics. In this research, four different contrast-enhanced staining solutions (CESS) including Iodine (I_2_) in Dulbecco’s modified eagle medium (DMEM), Phosphotungstic acid (PTA) in deionized water, PTA in ethanol, and Mercury II Chloride (HgCl_2_) in deionized water were used to visualize the tendon-to-bone interface using a combination of high resolution in-situ micro-computed tomography (micro-CT) imaging. The imaging was combined with DVC, nanoindentation, and quantitative 3D structural analysis to evaluate the effects of the CESS on the mechanical properties of the enthesis. The findings revealed significant alterations in mechanical behaviour and structural features of soft-to-hard tissue interfaces treated by CESS. The findings suggest that I₂ in DMEM provides a better balance between visualization and mechanical analysis. However, none of the CESSs completely preserved both structural and mechanical integrity.

**Supplementary Information:**

The online version contains supplementary material available at 10.1038/s41598-025-12729-6.

## Introduction

Tendon to bone insertion (TBI) or the enthesis is a non-homogenous, anisotropic, and multi-phase interface where tendon transitions into bone^1–3^. Fibrocartilaginous enthesis exhibits a complex hierarchical structure gradient of tissue zones transitioning from tendon to unmineralized and mineralized fibrocartilage before anchoring into bone^4^. Collagen fibres of different zones exhibit distinct mechanical and structural properties. The collagen fibres in the fibrocartilage zone are oriented parallel to the tendon or ligament axis, while in the mineralized fibrocartilage zone, the fibres become more oblique. In the calcified zone, the fibres insert into the mineralized matrix, anchoring the tendon or ligament to the bone^5,6^. This structural organization including collagen fibres, mineralized fibrocartilage, and their spatial arrangement enables the enthesis to mediate load transfer between soft and hard tissues while maintaining mechanical integrity during loading cycles^7,8^. Understanding the full-field microstructural and mechanical properties of this interface is essential for advancing the knowledge of musculoskeletal biomechanics and for developing biomaterials and therapeutic strategies to repair or regenerate damaged enthesis^9^. Several studies have investigated the structural and mechanical behaviour of tendon-to-bone interface^3,7,10–15^. Techniques such as confocal microscopy^16^ cryo-focused-ion-beam milling^17^ nanoindentation^18^ and atomic force microscopy^19^ despite their usefulness, have limitations when capturing three-dimensional (3D) information from complex samples and are unable to precisely measure the extent of disorganization or damage across the entire tissue caused by these techniques. Additionally, sample preparation may lead to anomalous measurements, for example the use of formaldehyde for tissue fixation can induce cross-linking, which in turn affects the tissue’s mechanical properties^20^. Finite element analysis^21^ provides a 3D mechanical measurement; however, accurately modelling the heterogeneous material properties and gradients across the tissue zones remains a significant challenge as precise information on the mechanical properties of the tissues related to the microstructure is incomplete^22^. Due to the strong relationship between structure and function in biological tissues, it is essential to visualize and quantitatively analyse tissue microstructure in 3D non-destructively^23^. High-resolution imaging techniques, such as micro-CT, have become invaluable tools for characterizing internal structure of materials^24,25^. Combining with in-situ mechanical testing, micro-CT applications have had significant advancements in the analyses of the structural changes of tissues subjected to mechanical loads^26–28^. The combination of digital volume correlation (DVC) with micro-CT provides a non-invasive experimental approach that enables the measurement of internal deformation and strain in materials by utilizing a sequence of 3D volumetric datasets, and calculates deformation vectors by analysing changes in grayscale structures^29,30^. Micro-CT applications in life sciences has been mostly limited to mineralized tissues because of the inherently low X-ray absorption of soft tissues such as tendons, ligaments, skin, fat, muscle, cartilage, nervous tissue, kidneys, cardiovascular tissues. To increase the absorption coefficient of soft tissues, x-ray attenuation contrast solutions containing atoms of high atomic numbers are used for contrast-enhanced (CE) micro-CT. This approach is particularly valuable in studies where both hard and soft tissues need to be observed as there is significant contrast differences between these tissues. Recent advancements in soft tissue micro-CT have made it possible to conduct 3D analysis of the microstructure within tendon, ligament, and enthesis organs^12,23,31^. Contrast agents like Iodine, PTA, and Phosphomolybdic acid (PMA) have been employed to enhance visualization of fibre orientations^32–34^. Iodine-based solutions have been extensively used as contrast solutions in micro-CT imaging due to their high affinity for biological tissues and ability to improve visualization of soft tissue microstructures. PTA is among the most used contrast agents for micro-CT/DVC applications of soft tissues^35–37^. However, the effects of these agents on tissue structure and mechanical properties are not fully understood . The accuracy of DVC calculations heavily relies on the presence of clear speckle patterns or distinct internal features within the microstructure to compute displacement and strain fields^38^. Any alteration in tissue properties, such as those introduced by contrast agents, may obscure these speckle patterns, leading to inaccurate DVC calculations and strain analysis. While contrast agents offer a promising solution for improving the imaging of soft tissues^33,39–46^ their application in in-situ mechanical measurements introduces potential chemical and mechanical effects on the tissues that must be thoroughly understood. For instance, some contrast agents such as Lugol’s iodine have been observed to cause tissue shrinkage^7,45,47^ or Hf-WD POM solution results in tissue swelling^23^. The volume changes induced by these agents can potentially influence volume-based DVC analysis, as the altered volume of the tissue may not accurately represent its natural configuration. These structural alterations are particularly critical in the enthesis, where the transition between mineralized and unmineralized regions has a heterogeneous structure and its function depends on the precise interplay between these regions.

Here we present the impact of four different CESS that have been used for micro-CT/DVC or enthesis mechanics research, including HgCl₂ in deionized water (H₂O), PTA in ethanol (EtOH) and in H₂O, on the visualization and mechanical characterization of the tendon-to-bone interface. I₂ in DMEM (due to its favourable preservation properties^48^) was selected due to its common use in medical imaging applications. While potassium iodide is commonly used alongside iodine to improve its solubility in aqueous solutions^23,49^ it promotes the formation of triiodide ions, which may increase tissue staining intensity but also contribute to acidification and potential structural deformation over time^50^. By integrating high-resolution in-situ micro-CT imaging, DVC, nanoindentation, and structural analysis, we aim to assess the influence of CAs on enthesis including tendon, fibrocartilage, and bone. We were able to determine how these agents affect tissue microstructure, strain distribution, global, and local mechanical properties, providing a comprehensive evaluation of their suitability for micro-CT/DVC of soft-to-hard tissue.

## Results

### TBI structure alterations by CESS

**Fig. 1:**
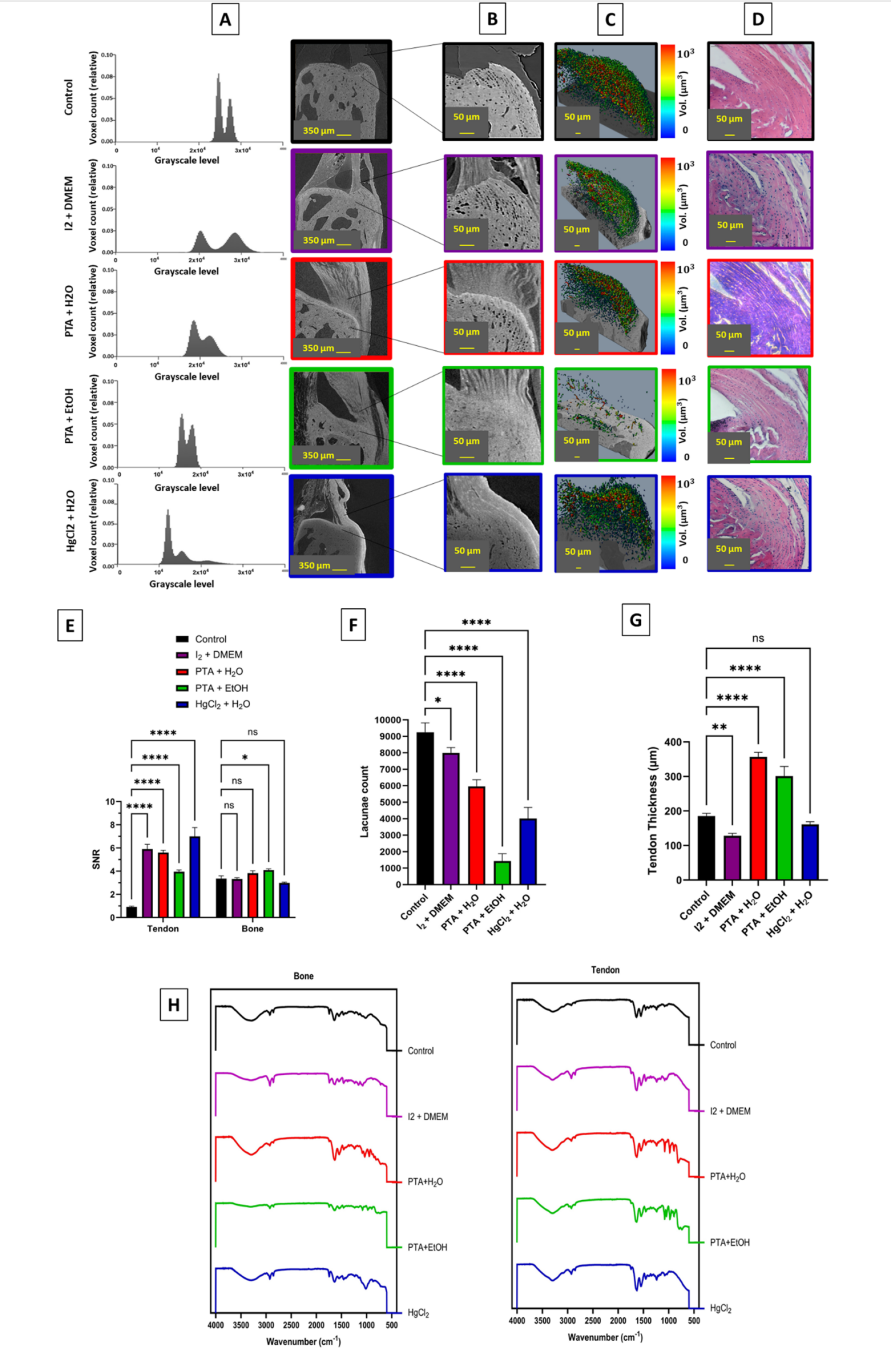
Structural alterations of tendon and bone tissue by different CESS. (**A**) Grayscale histograms for each CESS group are compared to evaluate the changes in tissue contrast caused by the contrast agents along with representative micro-CT 2D cross sections along mediolateral axis with scale bars of 350 μm. From top to bottom control, $\:{\text{I}}_{2}$ in DMEM, PTA in deionized water (H_2_O), PTA in ethanol (EtOH), and HgCl_2_ in H_2_O, showing the distribution of voxel counts at different grayscale levels. (**B**) Extracted enthesis volume for each treatment group, with scale bars of 50 μm. The images reveal the structural differences in tendon induced by various treatments, showing alterations in morphology. (**C**) 3D volume representation of lacunae at calcified fibrocartilage for each treatment group, coloured by lacunae volume from blue (50$\:{\mu\:m}^{3}$) to red (1000$\:{\mu\:m}^{3}$) (**D**) Histological images stained with H&E, highlighting the effects of the treatments on the structure. Scale bars are 50 μm. (**E**) SNR in tendon and bone regions in all groups. Contrast agents significantly enhanced SNR in the tendon, with HgCl_2_ in H_2_O showing the highest effect, while only minimal changes were observed in the bone region. (**F**) Quantification of lacunae count in bone tissue for each treatment group. (**G**) Measurement of tendon thickness for each treatment group. Significant differences are observed, with all treatments leading to thicker tendons except $\:{\text{H}\text{g}\text{C}\text{l}}_{2}$ solution showed no significant change. (**H**) FT-IR spectra of bone (left) and tendon (right) for each treatment group. Statistical significance is indicated as follows: ns = not significant, *p* < 0.05 = *, *p* < 0.01 = **, *p* < 0.0001 = ****.

To visualize the microstructure of the murine Achilles-to-calcaneus interface, we utilized high-resolution X-ray micro-CT imaging (1.3 μm/pixel) and CESS. Four CESSs were used for soft tissue visualization: I_2_ in DMEM, PTA in H_2_O, PTA in EtOH, and HgCl_2_ in H_2_O. An unstained sample was considered as the control. All groups were scanned under the following conditions: a voltage of 60 kV , 6 W power, a total of 1601 projections, and 5 s exposure time per projection. The histogram of grayscale intensities was extracted from the micro-CT tomograms for each CESS, and representative 2D micro-CT acquisitions are shown in (Fig. 1A and Sup. Figure S1). The leftward shift in the grayscale intensity histograms observed with the use of all CESS groups corresponds to darker peaks in the micro-CT images. This shift shows the changes in the X-ray attenuation of each sample due to the interaction between the contrast agents and tissue components. Two peaks indicate a clear threshold value for two phases. The left peak represents low-attenuation regions, e.g. soft tissue, and the right peak corresponds to higher-attenuation mineralized tissues. Grayscale intensity is directly related to the X-ray attenuation coefficient [μ=*ln* (*Io/Ix)/x*]^51^, where higher attenuation produces brighter voxels (high intensity gray level). However, the darker peaks in the histograms, despite the use of high-attenuation contrast agents, can be attributed to the alteration in the distribution of attenuation within the sample; for instance, the replacement of low-attenuation phases such as air and unstained soft tissues by contrast-enhanced Achilles tendon. To further evaluate the structural alterations in the calcaneal enthesis, a volume of interest (VOI) with dimension of 300 × 650 × 300 pixels (corresponding to 390 × 845 × 390 μm^2^, based on the isovoxel size of 1.3 μm) was extracted (Fig. 1B), which included the tendon, uncalcified fibrocartilage (UFC), calcified fibrocartilage (CFC), and bone. Significant changes in tendon visualization, thickness, and fibre arrangement were observed. The tendon thickness was quantified at a central cross-section through the mediolateral axis for each sample, specifically 50 ± 5 μm above the calcified fibrocartilage region for consistent analysis, to assess relative tendon shrinkage or swelling. The untreated sample had the thickness of 187 ± 12 μm, while treatment with I₂ in DMEM and HgCl_2_ in H_2_O resulted in noticeable tissue shrinkage, reducing tendon thickness to 126 ± 10 and 165 ± 10 μm, respectively. The unstained sample was scanned with 401 projections during a 50-minute X-ray exposure, which was optimized to minimize tendon dehydration that could otherwise affect the thickness measurements. The resulting tomogram was further enhanced through image processing techniques. In contrast, treatment with PTA in H_2_O and EtOH caused significant tissue swelling, with tendon thickness measured at 360 ± 15 and 280 ± 15 μm, respectively (Fig. 1B,G). PTA binds to collagen fibres and other ECM components to enhance the visibility and density of the low-contrast tissue^52,53^. The presence of water as a solvent facilitates hydration and swelling of the soft tissue. In contrast, while PTA in EtOH solution is known to induce dehydration and shrinkage, the PTA still binds to the ECM, leading to a denser and more compact appearance. Tendon fibres were visible in PTA solutions, while this was not the case in I_2_ and HgCl_2_ solutions. Additionally, the extracted lacunae from the CFC region (Fig. 1C,F) revealed significant differences in lacunae count. The unstained samples had an average of 9248 ± 570 lacunae, with lacuna size ranging from 50 to 1000 μm³. This number was significantly reduced in all other treated samples. PTA in EtOH resulted in the fewest lacunae (1424 ± 414), which may be due to ethanol creating a negatively charged environment, potentially aiding PTA penetration deeper into the mineralized tissue. PTA in EtOH also caused vague soft-to-hard tissue distinctions, making it more difficult to clearly differentiate between the tendon, fibrocartilage, and bone regions. I_2_ in DMEM showed the least reduction in lacunae count (7995 ± 325) compared to the control sample, and the lacunae were mostly filled in the superficial region of the calcified fibrocartilage, suggesting limited penetration or interaction of the stain in these areas. These differences in lacunae count impact the precision and reliability of DVC analysis by reducing the reference points to track tissue deformation accurately compared to native tissue. Histological analysis was conducted to assess the distribution of CESS within the enthesis structure. The sample stained with HgCl₂ solution exhibited a distinct, slightly darker coloration within the calcified fibrocartilage (Fig. 1D). The SNR calculation showed that all contrast agents significantly increased the signal-to-noise ratio in the tendon region compared to the control sample, with HgCl_2_ in H_2_O producing the highest enhancement, followed by I_2_ in DMEM, PTA in H_2_O, and PTA in EtOH. In the bone region, however, the majority of contrast agents did not significantly alter the SNR relative to the control, except for PTA in EtOH, which exhibited a slight but statistically significant improvement (Fig. 1E). Fourier-transform infrared spectroscopy (FT-IR) analysis was performed to examine the molecular bonding in samples treated with contrast agents (Fig. 1H). It was observed that samples treated with PTA showed new peaks ranging between 850 and 1200 indicating increased phosphate content^54^ and potential mineralization in tendon which are likely to contribute to higher stiffness and altered mechanical properties. Sample treated with HgCl_2_ solution showed only minor changes. However, based on micro-CT images, bright dots observed within the tendon fibres and cortical bone suggest the presence of mercury (II) ions, likely as precipitates within the material, without significant chemical bonding changes.

### Effects of different CESS on TBI global and local mechanical properties

**Fig. 2:**
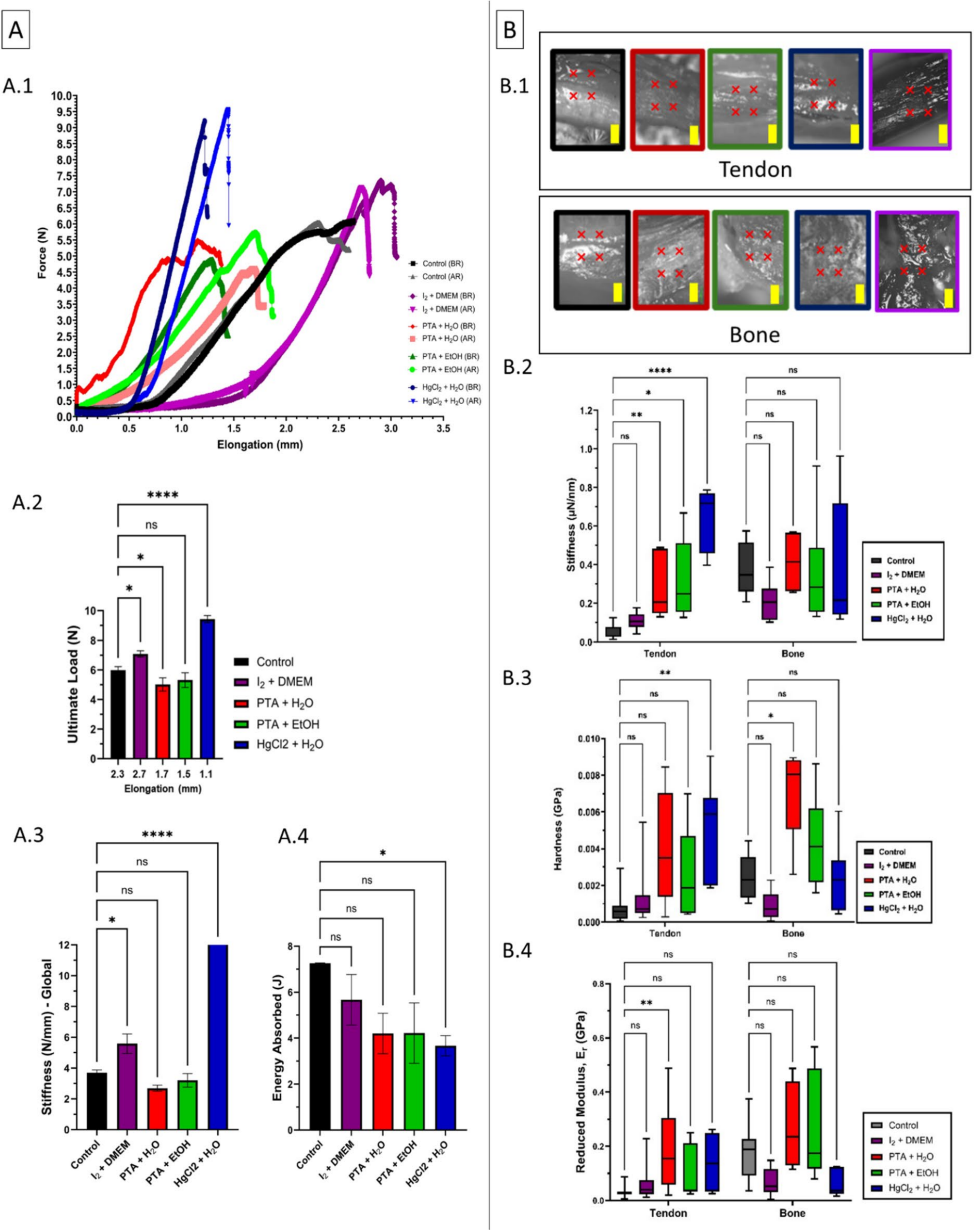
Global (**A**) and local (**B**) mechanical properties of TBI samples treated with different contrast agents. Data are reported as mean ± SD, *n* = 3/group. (**A.1**) Force-displacement curves before and after X-ray exposure. (**A.2**) Bar graph of ultimate load comparing samples’ ultimate load at ultimate elongation. (**A.3**) Global stiffness of samples in the linear region compared to untreated sample. (**A.4**) Energy absorption of samples. (**B.1**) Representative images of nanoindentation points (red crosses) of region of analysis on bone and tendon for each treatment. (**B.2**) Nanoindentation stiffness measurements of tendon and bone. Tendon stiffness is significantly higher in treated samples (except iodine) compared to the control, while bone stiffness shows no significant differences. (**B.3**) Hardness values of tendon and bone. (**B.4**) Reduced modulus of tendon and bone. Statistical analysis was performed using one-way ANOVA with comparison tests to the control group. Statistical significance is indicated as follows: ns = not significant, *p* < 0.05 = *, *p* < 0.01 = **, *p* < 0.0001 = ****.

To analyse the macro mechanical properties, in-situ micro-CT tensile testing was undertaken to obtain the force-displacement curves of murine Achilles tendon-to-bone samples stained with different contrast agents before and after 3-hours of exposure to X-rays (1601 projection and 5 s per projection) at a voltage of 60 kV. The results are plotted in (Fig. 2A). The control sample showed no notable differences in the force-elongation behaviour after radiation exposure. However, samples treated with I_2_ in DMEM, HgCl_2_ in H_2_O, and PTA in both H_2_O and EtOH exhibited slight alterations in their mechanical properties. Therefore, contrast agent-treated samples may experience subtle changes in their mechanical behaviour due to prolonged X-ray exposure. Based on the curves, the average ultimate load (mean ± SD) of samples treated with I_2_ in DMEM, HgCl_2_ in H_2_O, PTA in EtOH, PTA in H_2_O, and untreated samples were 7.09 ± 0.244 N, 9.43 ± 0.244 N, 5.317 ± 0.494 N, 5.023 ± 0.45 N, and 5.987 ± 0.244 respectively. Untreated tendon enters the plastic region after 23% elongation, while this occurs at 15% and 17% , in PTA in EtOH and H_2_O samples respectively, and for HgCl_2_ samples at 11%, and with I_2_ at 2.7%. Comparing the slope of the curves in the linear region; the sample stained with HgCl_2_ in H_2_O and I_2_ in DMEM has substantially higher stiffness (12.65 ± 0.64 N/mm, and 5.59 ± 0.63 N/mm respectively), while the sample stained with PTA either in EtOH (3.2 ± 0.44) or H_2_O 2.69 ± 0.19) showed no significant difference compared to the untreated sample stiffness (3.69 ± 0.19 N/mm). PTA-treated samples start linear behaviour at the toe region which accounts for up to 0.7% elongation. This indicates that PTA can impact the resting crimp angle of fibres, therefore allowing samples to enter the microscopic failure region of fibres at a lower elongation. The energy absorbed in untreated samples was significantly higher than HgCl_2_ in H_2_O and PTA in H_2_O, whereas PTA in EtOH had no significant difference compared to the control sample (Fig. 2A4).

Since the enthesis is a multi-zonal material, and its local mechanical properties of the different tissue zones cannot be locally assessed using standard uniaxial tensile testing, nanoindentation was employed to address this limitation (Fig. 2B). The samples were halved along the mediolateral axis, exposing the central region of the sample, and were examined in wet conditions to avoid the effect of dehydration on tissue mechanical properties. The data were normalized to account for potential pile-up effects during nanoindentation. Since the samples were not embedded, the likelihood of pile-up was higher^55^. To ensure the reliability of the measurements, any data where the ratio of the final indentation depth to the maximum indentation depth $\:({h}_{f}/{h}_{max})$ exceeded 0.7 (indicating significant pile-up) were excluded from the analysis^56–58^. Reduced modulus (E_r_, GPa) at bone region of the samples, showed no statistically significant differences across the CESS groups compared to the control sample (0.184 ± 0.11 GPa). Hardness (H, GPa) of bone samples; PTA in H_2_O (0.005 ± 0.003 GPa) shows a statistically significant increase compared to control group (0.002 ± 0.001 GPa), indicating that this treatment enhances the bone’s resistance to indentation at the nano-scale. The other agents showed no significant differences in bone hardness. The nanoindentation stiffness of treated bone samples showed no significant differences compared to the control (0.31 ± 0.16 µN/nm). Within the tendon region, HgCl_2_ in H_2_O showed the highest reduced modulus (0.38 ± 0.14 GPa) compared to all other treatments and the control sample (0.08 ± 0.02 GPa). HgCl_2_ + H_2_O significantly increases hardness (0.001 ± 0.0009 GPa), compared to the other treatments and the control (0.0007 ± 0.0002 GPa). All agents exhibited significantly higher stiffness than control samples (0.08 ± 0.02 µN/nm). The I_2_ in DMEM did not significantly impact the local mechanical properties of either the bone or the tendon in central regions. However, the samples treated with I_2_ in DMEM showed changes compared to the control sample during the tensile testing.

### CESS effects on Strain Distribution in micro-CT/DVC

**Fig. 3:**
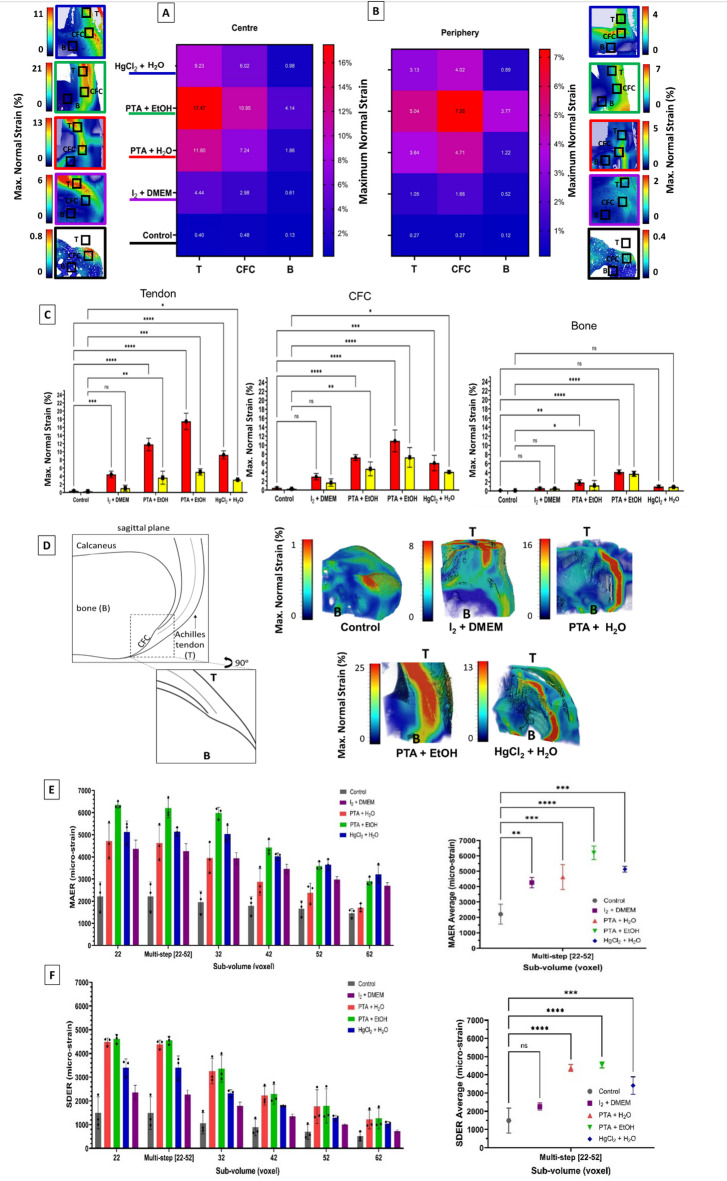
Strain distribution and DVC error analysis across treatments. (**A**,**B**) Heatmaps showing the maximum normal strain (%) distribution across central (**A**) and peripheral (B) regions of the tendon (T), calcified fibrocartilage (CFC), and bone (**B**) for all treatments, including control. Selected 2D slices in the mediolateral axis are displayed, representing the central and peripheral regions and highlighting the selected areas used to average the maximum normal strain. (**C**) Bar plots (red and yellow bars representing central and peripheral regions respectively) comparing maximum normal strain in tendon, CFC, and bone across treatments for central and peripheral regions. Significant differences are observed among treatments, with PTA in EtOH consistently exhibiting the highest strain values in all regions. (**D**) 3D strain maps illustrating strain localization patterns for each treatment, highlighting distinct differences in strain magnitudes and distributions induced by contrast agents. (**E**,**F**) DVC error analysis showing MAER (**E**), and SDER (**F**) across sub-volumes of varying sizes (22–52 voxels per side). Data are reported as mean ± SD, *n* = 3/group. Statistical analysis was performed using one-way ANOVA with comparison tests to the control group. Statistical significance is indicated as follows: ns = not significant, *p* < 0.05 = *, *p* < 0.01 = **, *p* < 0.0001 = ****.

We used DVC to evaluate the 3D full-field displacement and strain within the tendon to bone region by analysing the same 3D volume dimensions used for structural analysis, containing equal parts of tendon and bone in the tomograms. DVC error analysis was conducted using two consecutive scans acquired without mechanical loading as shown in (Fig. 3), DVC error was calculated using cubic sub-volumes ranging from 22 to 52 voxels per side (each voxel measuring 1.3 μm, corresponding to physical sizes of 28.6 μm to 67.6 μm per side). Mean absolute error (MAER) and standard deviation of error (SDER) decreased with increasing voxel size, as expected. For the control sample, a cubic sub-volume of 22 voxels per side exhibited an average MAER of 1300 micro-strain. However, the MAER and SDER significantly increased in all treatments due to the enhanced visibility of soft tissues and their potential movement caused by dehydration during the 3-hour interval between scans (Fig. 3E,F). This reflects reduced DVC accuracy of treated samples, particularly in smaller sub-volumes. For strain and load distribution analysis, samples were scanned under unloaded condition (reference dataset) and at 2 N based on acquired force-elongation curve of global tensile load. A multi-step DVC analysis was performed using sub-volumes ranging from 22 to 52 voxels per side based on error analysis. First, the maximum normal strain was calculated by correlating the reference dataset with the one under uniaxial tensile loading. Regions of interest measuring 150 × 150 μm were then selected within each tissue zone (tendon, calcified fibrocartilage, and bone) in both central and peripheral regions. The strain levels within these regions were averaged and presented to compare differences between treatments in (Fig. 3). The results demonstrate the strain across the tendon to the bone. Under applied load, the results of all stains revealed similar but distinct strain distribution trends across the tendon and CFC. In the central region, the tendon exhibited higher strain levels that gradually reduced toward the bone, which aligns with findings from previous research^7,14,15^. Conversely, in the peripheral region, the tendon strain was lower compared to the CFC. Within the CFC itself, the strain level at the centre was almost double that of the periphery, decreasing progressively toward the edges as well as a gradual reduction continuing towards the bone. Comparing the central and peripheral regions, strain within the tendon was consistently higher in the centre than in the periphery, with an almost 60% reduction in strain observed in treated samples and a 30% reduction in the control samples. This pattern of strain localization and dissipation was consistent across all treatments; however, the strain values were significantly higher in treated samples compared to the control. PTA in EtOH showed the highest strain values, reaching up to 25% at tendon, and 12% at CFC, while the control sample exhibited strain levels below 1% at CFC. The higher strain levels observed across all treated samples are partially attributed to the higher DVC error due to soft tissues dehydration and shrinkage.

## Discussion

DVC has become an essential technique for quantifying strain distribution and mechanical behaviour in biological tissues^59^. When paired with contrast-enhanced micro-CT, DVC enables detailed strain mapping and facilitates high-resolution microstructural features of soft tissues. However, the choice of contrast enhanced solutions can significantly influence mechanical and structural properties of the tissues, as well as the resolution of the acquired images. This study aimed to evaluate the impact of CESS on the structural and mechanical properties of the tendon-to-bone interface, particularly focusing on the murine Achilles-to-calcaneus interface. Using high-resolution X-ray micro-CT imaging, FT-IR spectroscopy, nanoindentation, and DVC, we assessed the effects of contrast solutions including I_2_ in DMEM, PTA in H_2_O and EtOH, and HgCl_2_ in H_2_O on tendon and bone. Table 1 summarizes the effects of various CESS on visualization, structural properties, and mechanical properties. The symbols used in the table indicate + for an increased effect, where the property was not significantly affected compared to the control, and for a reduced effect, where the property was significantly altered.

**Table 1:** Summary of the effects of contrast-enhanced staining solutions (CESS) on visualization, structural properties, and mechanical properties of the tendon-to-bone insertion. The symbols used in the table indicate “+” where the property was not significantly affected compared to the control, and “-” where the property was significantly affected.

CESS	Visualization and structural properties					Mechanical properties				
	SNR at tendon	SNR at bone	Lacunae count	Tendon thickness	Chemical bonding	Global stiffness	Local stiffness	DVC tendon	DVC bone	DVC accuracy
$\:{\mathbf{I}}_{2}$ in DMEM	+	+	+	−	+	−	+	+	+	−
PTA in H_2_O	+	−	−	−	−	+	−	−	−	−
PTA in EtOH	+	−	−	−	−	+	−	−	−	−
$\:{\mathbf{H}\mathbf{g}\mathbf{C}\mathbf{l}}_{2}$ in H_2_O	+	+	−	−	+	−	−	−	+	−

*SNR* signal-to-noise ratio, *DVC* digital volume correlation, *PTA* phosphotungstic acid, $\:\:{\text{I}}_{2}$ iodine, *DMEM* dulbecco’s modified eagle medium, hgcl_2_ mercury (II) chloride.

The contrast enhanced micro-CT revealed significant alterations in the tendon structure with different CESS treatments. Contrast agents were found to significantly influence the structural organization of the tendon-to-bone interface, particularly in terms of tissue swelling, shrinkage, and CFC lacunae alterations. Lugol’s iodine has proven effectiveness and popularity in conventional CT imaging. However, its application in micro-CT is associated with the potential for tissue shrinkage up to 70% for iodine solutions with high concentrations^33,60–62^. In this study, a low concentration of iodine (1%) is used in DMEM as an effective preservation medium^48^ to mitigate tissue shrinkage and improve structural integrity. The solution could fully penetrate in soft tissue after 24 h. The tendon thickness was reduced by 30% after 3 h exposure to x-ray. Based on altered mechanical properties acquired from tensile testing, and reduced area under the force-elongation curves before and after radiation, part of the tissue shrinkage is caused by soft tissues dehydration in lab-based micro-CT^61^. PTA is known for its ability to bind collagen, and with solvents such as ethanol has been reported to cause tissue dehydration and shrinkage^53,63^. In this research PTA solutions significantly increased the tendon thickness. FT-IR results revealed the presence of phosphate groups in PTA-stained samples, which contribute to potential mineralization leading to fibre swelling and altered mechanical properties. HgCl₂ exhibits a high affinity for thiol (sulfhydryl) groups in proteins^64^. Tendons are primarily composed of collagen, particularly type I collagen^65^. Col I does not contain significant amounts of thiol groups^66^. However, thiol groups are present in other minor proteins or enzymes associated with the ECM of tendons^67^. This contrast agent, as observed in micro-CT tomograms, predominantly precipitated non-homogeneously within the ECM, causing contraction in the tendon and significantly reducing its thickness. In the bone region, it predominantly aggregated at the bone surface including fibro chondrocyte lacunae, as observed in previous research^12^ and could not penetrate further into the tissue. This resulted in significant brightness at the bone surface region, reducing contrast within other regions of the bone. The lacunae in the calcified fibrocartilage further highlighted the structural impact of these contrast agents. PTA in EtOH resulted in the greatest reduction in lacunae count, due to deeper penetration into the tissue. In contrast, I₂ in DMEM showed the least change, with lacunae mostly filled in the superficial regions of the calcified fibrocartilage, suggesting limited penetration of the contrast agent into the mineralized tissue. This discrepancy in lacunae count can affect the precision of DVC analysis by reducing the number of reference points for tracking tissue deformation. Lacunae play an important role in modulating strain distribution^68^ and their structural deformation caused by contrast agents can significantly affect the strain analysis. This quantification is important when analysing mechanical properties through contrasted enhanced imaging and volume-based analysis at soft to hard tissues.

The mechanical behaviour of TBI was markedly influenced by the CESS. Samples treated with HgCl₂ in H₂O exhibited increased stiffness and hardness due to molecular aggregation and the precipitation of mercury ions within the ECM. PTA-treated samples, on the other hand, demonstrated reduced stiffness and earlier plastic deformation in both tendon and bone. Although PTA can induce mineralization, the slow penetration and prolonged exposure of samples in solutions lead to significant tissue swelling, ultimately resulting in a loss of stiffness.

DVC accuracy analysis revealed a significant increase in error calculations, partly attributed to tendon image resolution. This was not the case for the control sample. Soft tissues inherently dehydrate and shrink at room temperature and under conditions leading to partial or complete dehydration. In DVC, the alignment of two gray level volumes, relies on the preservation of image texture^30^. Following X-ray exposure, it is evident that deformations caused by dehydration associated with acquisition time are exacerbated in soft tissues, leading to higher DVC errors in zero-strain experiments due to voxels displacements. PTA in EtOH exhibited the highest error, as ethanol promotes greater dehydration within the tissue. These deformations, occurring prior to the application of load during DVC measurements, result in elevated strain levels in soft tissue during DVC strain analysis. DVC results produced full 3D internal strain maps at tendon to bone insertions and CFC regions with all treatments presenting similar trends in strain.

Among the CESSs, I₂ in DMEM emerges as the most favourable agent with the highest number of positive scores across the categories. It positively enhanced the SNR and did not significantly affect the calcified fibrocartilage pore structure, which is critical for DVC correlation values. However, similar to the other agents, it negatively affected the tendon thickness. None of the tested CESSs fully preserved these microstructural features. I_2_ in DMEM had the least effect on DVC results with no significant effect on most of regions except for the central regions of tendon. I_2_ in DMEM negatively affected (*p* < 0.05) global stiffness while preserved local stiffness. Its overall performance across mechanical and visualization metrics makes it a relatively better choice for applications prioritizing DVC strain mapping in soft tissues.

A limitation of this study is the absence of quantitative surface roughness analysis prior to nanoindentation. However, care was taken to select consistent regions visually smooth and flat under the integrated optical microscope. The load control was selected to allow consistent application across both soft and mineralized tissues. As expected, the indentation depths differed between tendon and bone. The nanoindenter (Bruker Hysitron Ti Primier) has a displacement sensitivity of 2 nm, and all force-displacement curves showed stable behaviour without noise. Although the indentation depth in bone was lower than in tendon, it still reached up to 100 μm, which is well above the displacement sensitivity threshold. While we acknowledge that load control results in variable depth, it was deemed appropriate for this comparative study, where the primary aim was to assess relative changes in local mechanical response between treatment groups.

Another limitation of this study is the use of only four nanoindentation points per sample. Although points were selected consistently across anatomical regions, the limited number may reduce statistical power and increase variability. Future work incorporating a greater number of indentation sites are recommended to strengthen these findings.

Future studies could focus on optimizing contrast-enhanced staining protocols to further minimize tissue alteration during imaging. Buffered staining solutions or alternative agents with lower osmotic and chemical effects could offer improved preservation of microstructure and mechanical integrity. Additionally, implementing cryo-micro-CT, could allow high-resolution structural analysis while maintaining native tissue hydration and minimizing fixation-related artifacts. Combining cryogenic preservation with advanced DVC analysis could provide more accurate assessments of local strain distribution across the tendon-to-bone interface.

## Materials and methods

### Sample preparation

Achilles tendon to Calcaneus samples (*n* = 68) were dissected from 34 wild-type 8-month-old male mice sourced from University of Portsmouth. Ethical approval was granted by the School of Electrical and Mechanical Engineering, University of Portsmouth, under Ethics number TETHIC2022-104588. Mice were euthanized by cervical dislocation. All experimental protocols were approved by University of Portsmouth. All methods were carried out in accordance with relevant guidelines and regulations and are reported in accordance with ARRIVE guidelines. The samples were cut from the tendon-to-muscle junction, and the skin, tibia, and connective tissues surrounding the Achilles tendon were removed under a dissecting microscope (Vision Engineering, UK). Samples were stored frozen at -20 °C until used. Although direct evaluation of frost damage was not conducted in this study, previous investigations have demonstrated that freezing tendon tissue at − 20 °C does not significantly affect its microstructure or mechanical properties^69^. PTA (Merck, Germany) in H₂O, PTA in EtOH, HgCl₂ (Merck, Germany) in H₂O, and I₂ (Merck, Germany) in DMEM (Thermo Fisher Scientific, USA) were selected as CESS and compared to untreated (control) specimens. For PTA-stained samples in EtOH, samples were immersed in 100% EtOH for one hour before transferring to a 1%v/w PTA solution in 75% EtOH for 3 days. PTA-stained samples in H₂O were immersed in a 1%v/w PTA solution for 3 days without prior ethanol immersion. 1% v/w HgCl₂-treated samples were immersed in water for 24 h. 1% v/w I₂ was dissolved in 3% ethanol in DMEM solution.

### Micro-CT dataset acquisition and post-processing

To characterize the microstructure of the enthesis before and after tensile testing, micro-CT scans were performed using the Zeiss XRadia Versa XRM 610 system (Carl Zeiss, Germany). A total of 1601 projections with 5-second exposure time per projection were acquired at 60 kV voltage and 6 W power. Optical magnification of 4X was used, with a 21 mm source-to-specimen distance and an 85 mm specimen-to-detector distance, resulting in a pixel size of 1.3 μm. Two tomograms were obtained for DVC analysis (*n* = 3/group), pre- and post-loading. Pre-load scans were used for structural analysis such as lacunae count and tendon thickness. For tendon thickness measurement the unstained control sample (*n* = 3) was scanned with a reduced number of projections (401 vs. 1601 for stained samples) to minimize total scan time and reduce dehydration-related volume shrinkage. The unstained tendon has inherently low X-ray contrast, its boundaries appear as diffuse shadows rather than clearly defined structures. In this context, image resolution was less critical than maintaining realistic tissue volume. The reduced scanning time helped preserve the hydrated geometry of the tendon, which in turn allowed shadow-based thickness measurement with minimal distortion. Reconstruction of micro-CT datasets was performed using the Scout and Scan Control System (Zeiss Medical Technology). 16-bit images/datasets were imported into Avizo software, version 2021.2 (Thermo Fischer Scientific, USA) for post-processing. Datasets were manually registered using rigid registration, and a VOI with dimensions of 300 × 650 (refers to mediolateral axis) x 300 voxels were extracted focusing on the tendon-bone interface. Tendon thickness was measured from a consistent mediolateral central section, located 50 ± 5 μm above the calcified fibrocartilage region, using Avizo software, version 2021.2 (Thermo Fischer Scientific, USA). Anatomical consistency was ensured by selecting comparable cross-sectional planes across all samples, using identifiable structural landmarks of the insertion site such as protrusion and its depth. To assess image quality of contrast-enhanced images, five pairs of patches were randomly selected across the field of view, once calculated for the bone tissue and background, and once for the tendon tissue and background. The SNR was calculated based on the formula $\:SNR=\:\frac{{\mu\:}_{tissue}-\:{\mu\:}_{air}}{\sqrt{{\sigma\:}_{tissue}^{2}-\:{\sigma\:}_{air}^{2}}}$ Where, $\:{\mu\:}_{tissue}$ and $\:{\mu\:}_{air}$ represent the mean intensities of patches corresponding to bone or tendon, and background regions, respectively. $\:{\sigma\:}_{tissue}$ and $\:{\sigma\:}_{air}$ denote the standard deviations of pixel intensities within the same bone or tendon, and background patches.

### Lacunae extraction

Datasets were imported in XamFlow software, version 1.19.0.0 (Lucid AG. Switzerland). First, the intensity histograms of tomograms were matched. Pores were extracted by reverse thresholding. A morphological operation known as opening was performed to separate connected binarized lacunae and enable their numbers to be counted and labelling of the individual lacunae. The lacunae volume was restricted to 50–1000 μm^2^. Non-lacunar structures such as small voids or irregular features located near the CFC and adjacent trabecular bone, including small trabecular spaces or channels were not included in the analysis. Lacunae were automatically identified by XamFlow software, version 1.19.0.0 (Lucid AG. Switzerland).

### Histology

Samples were fixed in 10% formalin for 1 day, decalcified in ethylenediaminetetraacetic acid (EDTA) for 2 weeks, and dehydrated in ethanol concentrations of 50%, 75%, and 100% (3 h each). Samples were cleared in xylene (3 cycles, 9 h total), infiltrated in paraffin twice (6 h at 60 °C), and embedded in paraffin wax (Leica, Germany). Serial Sect. (8 μm) were prepared using a microtome (Leica, Germany), baked on slides for 30 min at 60 °C, and stained with Hematoxylin (CellPath, UK) and Eosin Y (Merck, Germany). Images were captured using an LED digital microscope (Leica, Germany).

### FT-IR measurement and spectral collection

Tissue samples, including tendon and bone, were sectioned into pieces of approximately 1–2 mm in size. FT-IR combined with attenuated total reflectance measurements were conducted using an FT-IR spectrometer (Nexus, Thermo Fisher Scientific, USA). Each sample was measured over the spectral range of 4000 to 400 cm^−1^, with transition mode and resolution of 4 cm^−1^. For each sample, 3 repeat spectra were recorded, and the resulting spectrum was taken as the average of three measurements. A total of 32 scans were collected per sample. A background spectrum was collected before each measurement. Spectral data were analysed using the FT-IR spectrometer’s software (Omnic, Thermo Fisher Scientific, USA).

### Nanoindentation

Quasi-static nanoindentation (Bruker Nano indenter, USA) using a conical diamond tip, with 20 μm nominal radius and 1140 GPa Young’s modulus (Bruker Hysitron Ti Primier, USA), was conducted at tendon and bone regions of murine Achilles to calcaneus bone (*n* = 3/group). Specimens were hydrated while testing and a load of 5 µN was applied at load rate of 1 µN/s. The samples were examined in wet conditions using PBS to avoid the effects of dehydration on the tissue. A custom-made holder (Sup. Figure S2) was designed to accommodate the thickness difference between the tendon and bone, securing the tendon-to-bone specimen. The sample was halved longitudinally using a sharp blade to create a flat surface, exposing the central region of the sample. This ensured that the same area was analysed across specimens, providing consistency and comparable mechanical properties, as these properties may vary locally. Since the sample was not embedded, the trabecular bone structure remained unstable for indentation. For each sample, indentation points were consistently selected in the cortical bone near the tendon-bone interface and in the central region of the tendon. A total of four indentation points were manually placed on visually smooth and flat surfaces, as identified under an optical microscope integrated with the nanoindentation system. These locations were chosen based on optical brightness and surface uniformity. The load-depth curves obtained were analysed using the Oliver-Pharr method^57^ to determine the indentation hardness, stiffness, and the reduced modulus.

### Global mechanical properties of samples

Samples (*n* = 3/group) were prepared to a 20 mm length and clamped onto the tensile platens of the CT500 (Deben, UK) loading stage. The muscle-tendon junction (MTJ) was clamped to the mobile jaw, and the bone to the fixed jaw, maintaining a 10 mm distance between the jaws. The tensile axis of the tendon was aligned with the calcaneus axis. To prevent rupture at the MTJ during loading, the MTJ was secured with adhesive. Cyanoacrylate adhesive was used to attach sandpaper to both ends of the samples (5 mm).Tensile testing to failure was conducted to determine the global mechanical properties of the samples. Stained and unstained samples underwent uniaxial tensile testing until failure before and after 3 h of X-ray exposure. To facilitate comparison, global stiffness (N/mm) was calculated from the linear range of the force-displacement curve (∆F/∆L), while energy absorption (J) was determined by integrating the area under the curve up to ultimate load and elongation.

### Tensile test for DVC strain map

In-situ micro-CT tensile testing was performed using the Deben CT500 (Deben, UK) under load control. Treated samples (*n* = 3/group) and untreated samples (*n* = 3) were tested. A maximum load of 2 N, determined from the linear range of tensile failure curves, was applied at a motor speed of 0.1 mm/min with a 100 ms sampling time. Force-displacement curves were recorded after each test.

### Digital volume correlation and uncertainties

DVC measured the full-field 3D strain at the interface. Maximum normal strain was calculated from deformed samples. Processed datasets were imported into DaVis software (10.1.5 LaVision, UK) for DVC analysis. A multi-step DVC processing scheme was applied using cubic sub-volumes ranging from 22 to 52 voxels per side (i.e., 22 × 22 × 22 to 52 × 52 × 52 voxels). Each voxel measured 1.3 μm along the X, Y, and Z axes, corresponding to isotropic (isovoxel) spacing and physical sizes ranging from 28.6 μm to 67.6 μm per side. A 0% overlap was used between sub-volumes, and rigid body movements were removed prior to strain calculation. Strain maps were overlaid on micro-CT datasets in Avizo software, version 2021.2 (Thermo Fischer Scientific, USA) for visualization. To analyse DVC uncertainties, two consecutive tomograms were acquired under zero-strain conditions. Uncertainty analysis was conducted using single-step DVC with sub-volumes ranging from 22 to 52 voxels per side, each voxel measuring 1.3 μm. Six deformation components (εxx, εxy, εxz, εyy, εyz, εzz) were extracted to calculate the mean absolute error and standard deviation of error^70,71^.

### Statistical analysis

Statistical analysis was conducted using GraphPad Prism 9.4.1 (SD, USA). Data were expressed as mean ± standard deviation (SD), with P values < 0.05 considered statistically significant. Comparisons test was assessed using one-way or two-way ANOVA to compare each contrast agent (structural and mechanical properties), and the control group (*n* = 3 replicates per group).

## Electronic supplementary material

Below is the link to the electronic supplementary material.

Supplementary Material 1

## Data Availability

The full data is available from the corresponding authors on request.
