# Supplementary material for: Evaluating the impact of contrast agents on micro and nano mechanics of soft-to-hard tissue interface
Source: Sci Rep. 2025 Aug 2;15:28267. doi: 10.1038/s41598-025-12729-6 (PMC12317997; doi:10.1038/s41598-025-12729-6)
Supplement: Supplementary file 1 — Supplementary Material 1 [file 41598_2025_12729_MOESM1_ESM.docx]

Supplementary materials for Evaluating the Impact of Contrast Agents on Micro and Nano Mechanics of Soft-to-Hard Tissue Interface

Atousa Moayedi^1*^, Katerina Karali^1^, Markus Boese^3^, Jurgita Zekonyte^1^, Jovana Radulovic^1^, Gordon Blunn^2^

^1^ School of Electrical and Mechanical Engineering, University of Portsmouth, United Kingdom

^2^ School of Medicine, Pharmacy and Biomedical Sciences, University of Portsmouth, United Kingdom

^3^ Carl Zeiss Microscopy GmbH, Germany

**Tendon to bone microstructure**


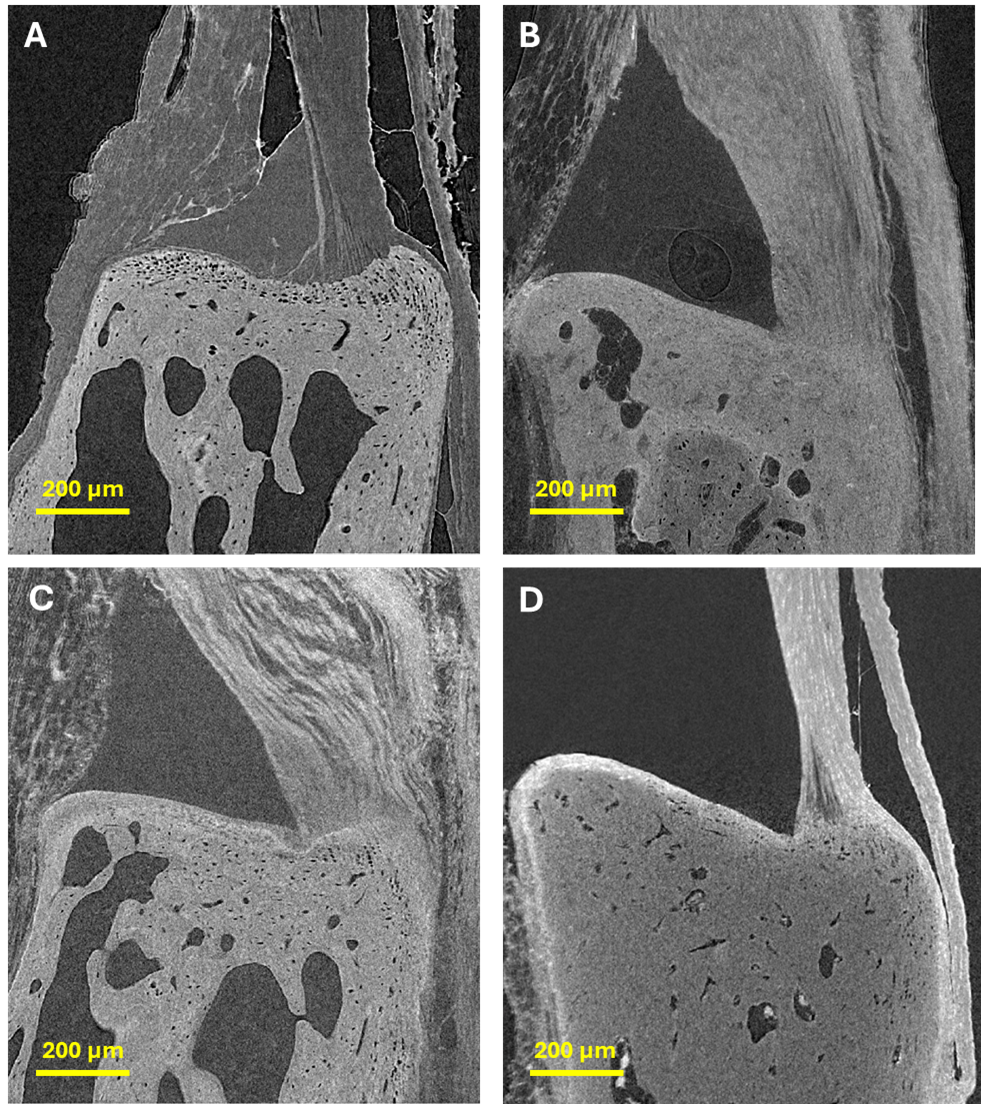


**Sup. Figure S1: Representative sagittal micro-CT slices showing the murine Achilles tendon to calcaneus under different contrast-enhanced staining conditions.** **(A)** I₂ in DMEM, **(B)** PTA in ethanol, **(C)** PTA in H₂O, and **(D)** HgCl₂ in H₂O. These high-resolution scans (pixel size: 1.3 μm) depict the insertion of tendon fibres into the calcaneus and the precise anatomical location of tendon insertion on the calcaneus. Tendon fibres appear to align with rows of lacunae in the CFC, suggesting structural continuity across the interface.

**Sample holder for nanoindentation test**


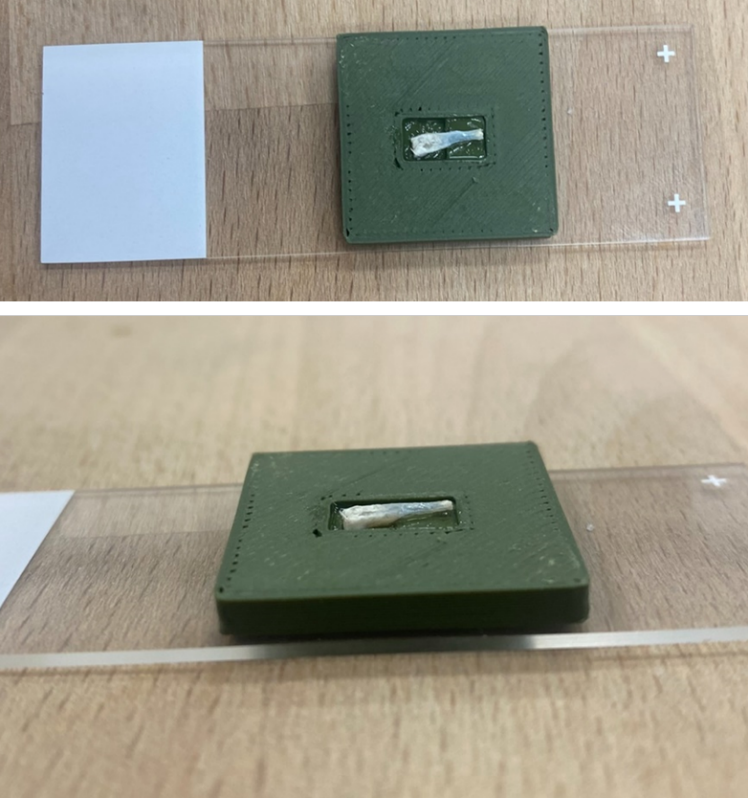


**Sup. Figure S2: Sample preparation for nanoindentation in wet conditions.** A half-elevated holder was designed to accommodate the thickness difference between the tendon and bone, securing the tendon-to-bone specimen. The sample was glued to the holder and halved along the mediolateral axis using a sharp blade to create a flat surface, exposing the central region of the sample. This ensured that the same area was analysed across specimens. A few drops of PBS were added to keep the sample hydrated.
